# Proton transfer during reduction of the catalytic metallo-cofactors of the three nitrogenase isozymes

**DOI:** 10.1039/d5sc05488e

**Published:** 2025-09-12

**Authors:** Roman Davydov, Dmitriy A. Lukoyanov, Derek F. Harris, Dennis R. Dean, Lance C. Seefeldt, Brian M. Hoffman

**Affiliations:** a Department of Chemistry, Northwestern University 2145 Sheridan Road Evanston Illinois 60208 USA bmh@northwestern.edu r-davydov@northwestern.edu d-lukoyanov@northwestern.edu; b Department of Chemistry and Biochemistry, Utah State University Logan Utah 84322 USA lance.seefeldt@usu.edu derek.harris@usu.edu; c Department of Biochemistry, Virginia Polytechnic Institute and State University Blacksburg Virginia 24061 USA deandr@vt.edu

## Abstract

Nitrogenase catalyzes biological nitrogen fixation, the conversion of atmospheric N_2_ into bioavailable ammonia. The three nitrogenase isozymes—Mo-nitrogenase, V-nitrogenase, and Fe-nitrogenase—utilize catalytic cofactors distinguished by their metal composition (Fe_7_M, M = Mo, V, or Fe; denoted FeM-co). Their catalytic cycles involve stepwise addition of 8[e^−^/H^+^] to FeM-co, generating intermediates designated E_*n*_, where *n* is the number of [e^−^/H^+^] delivered. The electron-transfer has been extensively characterized, but the proton delivery has not. Here, we investigate [e^−^/H^+^] delivery during early-stage conversions, primarily E_0_ → E_1_(H), for each of the three nitrogenases, using as reductants γ-ray-generated thermolyzed, mobile electrons at 77 K, and radiation-generated solvent radicals during subsequent annealing to higher temperatures. Our results show E_0_ → E_1_(H) conversion differs among the three MFe-proteins. The FeMo-co of MoFe-protein accepts an electron (ET) during 77 K γ-irradiation, but proton transfer (PT) to generate E_1_(H) is only enabled by conformational or thermodynamic activation upon cryoannealing to ∼200 K(ET/PT). For VFe-protein, E_1_(H) forms during annealing at-and-above 210 K by electron-transfer to FeV-co from radicals through proton-coupled electron transfer (PCET), which too is enabled by activated proton transfer. FeFe-protein differs in directly exhibiting delivery of protons at 77 K, which together with the mobile electrons react to form E_1_(H). This could well occur by PCET at 77 K, but does not preclude the possibility of sequential 77 K electron/proton transfer (ET/PT). In addition, 450 nm photolysis reveals the E_1_(H) state of FeV-co, like that of FeFe-co, contains a hydride bound to a formally oxidized cofactor. The mechanistic differences observed here provide a contribution towards understanding the sources of catalytic differences among the three nitrogenase isozymes.

## Abstract 2

Nitrogenase catalysis involves stepwise addition of 8[e^−^/H^+^]. Electron-transfer has been extensively characterized, but proton delivery has not. We investigate early-stage [e^−^/H^+^] delivery for the three nitrogenase isozymes.

## Graphical abstract

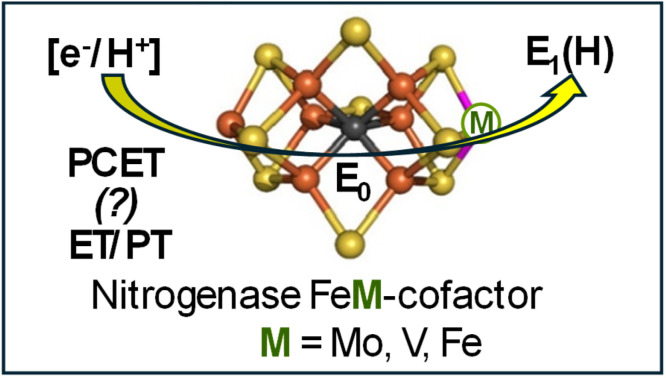

## Introduction

**Fig. 1:**
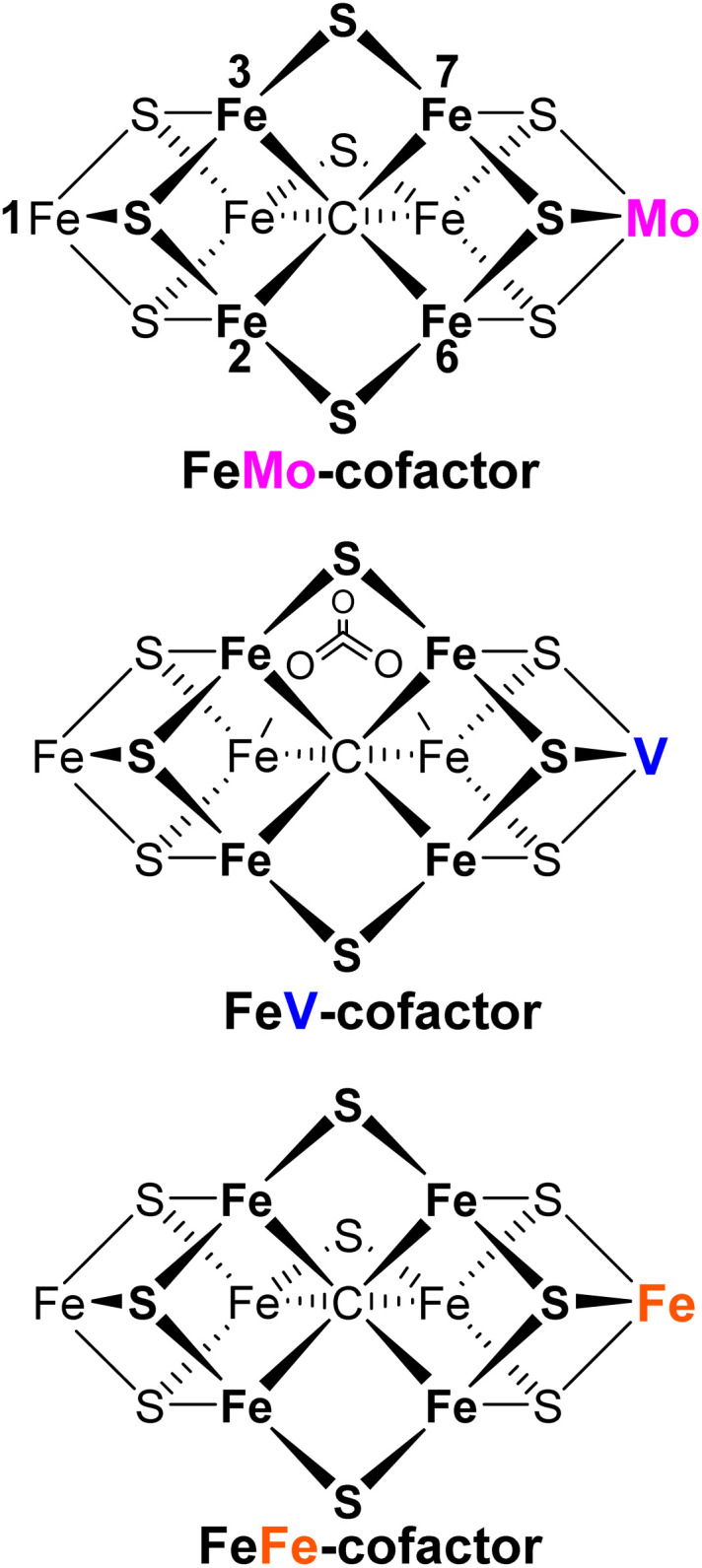
FeM-cofactors.^1,2^

**Scheme 1:**
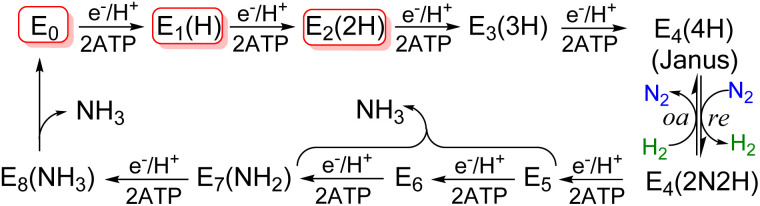
Simplified Lowe-Thorneley scheme for N_2_ reduction by nitrogenase. Red boxes indicate intermediates studied here; brace indicates possible steps where the first NH_3_ is released.

All nitrogen (N) in living organisms derives from dinitrogen (N_2_) in the atmosphere, and Nature's solution for converting the stable N_2_ molecule into biologically available ammonia (NH_3_) is the enzyme nitrogenase. There are three nitrogenase isozymes, all of which work by a common mechanism that incorporates a limiting stoichiometry, catalysis occurs at a complex metallo-cofactor, denoted here as FeM-co, with 7 Fe ions and an additional metal-ion, M, where M = Mo (FeMo-co) in the most-abundant Mo-isozyme, M = V (FeV-co) in the V-isozyme, and M = Fe (FeFe-co) in Fe-isozyme, with structures of these catalytic cofactors schematized in (Fig. 1).^1,2^ As indicated in the simplified Lowe-Thorneley scheme (Scheme 1), during catalysis, the stepwise addition of the 8[e^−^/H^+^] to an isozyme by its cognate electron-delivery Fe protein generates intermediates designated E_*n*_(L), where *n* is the number of [e^−^/H^+^] that have been delivered to the as-isolated resting state, simply denoted E_0_, and L signifies bound species.^3,4^ We have shown that N_2_ binding occurs after accumulation of *n* = 4[e^−^/H^+^], and is driven by the concomitant reductive elimination of H_2_.^4–7^ In the electron-accumulation phase, E_0_ → E_4_, the rate of transition between successive intermediates is controlled by the kinetic events of a so-called Fe protein cycle that involves Fe-protein reduction/e^−^ delivery coupled to binding/hydrolysis of ATP plus binding-to and release-from MoFe, eqn (1).^8–11^

1 N_2_ + 8[e^−^/H^+^] +16ATP → 2NH_3_ + H_2_ +16ADP +16Pi

Recent progress has provided important insights into the properties of multiple E_*n*_-states. The *n* = even states of the MoFe-protein FeMo-co are paramagnetic and thus can be studied by electron paramagnetic resonance (EPR) and electron-nuclear double resonance (ENDOR) spectroscopies to provide unprecedented insights into the cofactor in these states. Focusing on the early stages of the catalytic cycle, the E_2_(2H) intermediate, formed by the delivery of 2[e^−^/H^+^], contains a single Fe-bound hydride and a proton assigned as bound to a cofactor sulfide.^12^ The E_4_(4H) state has been trapped and found to contain two iron-bridging hydrides, with two protons assigned as bound to two cofactor sulfides.^13,14^

Whereas FeMo-co is paramagnetic in the E_0_ and even-*n* states, FeV-co^15^ and FeFe-co^16^ are paramagnetic in the odd-*n* states. The E_1_(H) state of FeFe-protein and the E_1,3_(1,3H) state of the VFe-protein, so labelled because it had not been established whether *n* = 1 or 3, have been trapped and studied by EPR.^15,16^ The EPR spectrum of E_1_(H) FeFe-cofactor has *g* = [1.965, 1.928, 1.779].^16^ At 12 K, this state is photoactive to 450 nm light,^17^ showing conversion to a new and likewise photoactive *S* = 1/2 conformer (denoted E_1_(H)*) with *g* = [2.009, 1.950, 1.860], which relaxes to E_1_(H) at temperatures above 145 K. Illumination of these two *n* = 1 states at cryogenic temperatures forms a photostationary state, with an H/D kinetic isotope effect of 2.4 accompanying the E_1_(H)/E_1_(H)* photointerconversion. These observations indicate that the addition of the first e^−^/H^+^ to the FeFe-co produces an Fe-bound hydride, not a sulfur-bound proton.^17^ As a corollary, the cofactor metal-ion core of E_1_(H) is formally characterized as being one-electron oxidized relative to the resting state. It was proposed that this behavior applies to all three nitrogenase isozymes.

In this report, we combine the results of 77 K γ-ray cryoradiolysis and subsequent annealing EPR measurements of each MFe-protein with those of earlier Mossbauer^18^ and X-ray spectroscopic measurements,^19^ to investigate electron/proton delivery to the FeM-cofactors during the E_0_→E_1_(H) conversion. Electron delivery to a FeM-cofactor during catalytic nitrogenase turnover with the Fe protein at ambient temperatures is an extraordinarily complex process, as shown for the MoFe protein.^20–23^ However, until rather recently,^19,24,25^ the corresponding delivery of protons was only assumed for charge balance and to fill the need for protons in substrate reduction, and little is still known about the details of proton delivery. To further address this issue, for each isozyme we here use 77 K γ-ray cryoradiolysis to bypass the Fe-protein cycle, which thereby allows us to monitor electron and proton delivery to the cofactor itself, and to ask by which pathway an electron and proton add to the cofactor during the E_0_ → E_1_(H) conversion: (i) electron transfer followed by proton transfer, ET/PT; (ii) the reverse, proton transfer followed by electron transfer, PT/ET; or (iii) concerted proton-coupled electron transfer, PCET, which in the limiting case of concerted proton-electron transfer is denoted CPET.^23,26–33^

To initiate the E_0_ → E_1_(H) conversion, mobile electrons are generated by 77 K γ-ray cryoradiolysis; its completion through the formation of E_1_(H) is then observed, either directly at 77 K or during cryoannealing of the frozen solution at successively higher temperatures. Proton transfer in Mo- and V-proteins is only enabled by conformational or thermodynamic activation during cryoannealing at higher temperatures, typically ∼200 K and above, as seen in the O_2_-activating enzyme nitric-oxide synthase.^34^ In contrast, these measurements show that in FeFe-protein active-site proton-delivery occurs at 77 K without need for such activation, behavior similar to that of active-site proton delivery networks in the O_2_-activating enzymes cytochrome P450cam and heme oxygenase, which function at cryogenic temperatures of 77 K and even below.^35,36^ In addition, 450 nm photolysis of E_1_(H) of VFe-protein indicates that it, like E_1_(H) of FeFe-protein,^17^ contains a bound hydride, and thus a cofactor that is formally oxidized.

## Materials and methods

### Reagent and general procedures

All reagents were obtained from Sigma-Aldrich, Fisher-Scientific or BioRad and used without further purification. Argon and dinitrogen gases were purchased from Air Liquide America Gases (Plumateadville, PA). Manipulation of protein and buffers was done anaerobically in septum sealed serum vials and flasks using vacuum Schlenk line under an argon atmosphere. Gas transfers were made using gastight syringes.

### Bacterial strain growth, protein purification, and sample preparation

MoFe, VFe, and FeFe-proteins were expressed and purified from respective *Azotobacter vinelandii* strains DJ2102 (*nifD*^Strep^), DJ2254 (*vnfD*^Strep^), and DJ2387 (*anfD*^Strep^) by Streptactin-based affinity chromotography, as previously described.^37^ Proteins were prepared at 200 μM in 200 mM MOPS, 100 mM NaCl, pH 7.3 with 20% glycerol (v/v) and 50 mM sodium dithionite. 350 μL of the sample was added to a 4 mm quartz EPR tube and frozen in a pentane slurry.

### Cryoreduction

Cryoreduction of samples at 77 K in liquid nitrogen was performed in a^60^Co γ-ray source (rate is 1 Mrad hour^−1^). Total dose was 3.5 Mrad, which we found to optimize the generation of singly-reduced metal centers.^34–36^

### EPR spectroscopy and photolysis

EPR spectra were recorded on X-band Bruker ESP-300 spectrometer equipped with Oxford Instruments ESR 900 continuous liquid He flow cryostat; conditions in all spectra, microwave frequency, *ν*_MW_ = 9.364 GHz; Mod Amp = 10 G. *In situ* photolysis of samples at cryogenic temperatures was performed through waveguide beyond cutoff attached to the cavity front face, using a Thorlabs Inc. PL450B, 450 nm Osram Laser Diode with power adjusted to 80 mW. EPR simulations: EasySpin.^38^

## Results and discussion

### Cryoreduction/annealing of nitrogenase and proton and electron transfer

We here explore the conversion E_*n*_ → E_*n*+1_ (*n* = 0, 1) through [e^−^/H^+^] delivery in frozen MFe-proteins. In part this involves thermolyzed, mobile electrons that are produced by 77 K γ-irradiation of solvent (cryoreduction). In addition, we find that during cryoannealing to higher temperatures, typically *T* ≥ 145 K, reduction can occur by electron-transfer from the organic radicals that are generated by solvent irradiation, and which persist in the frozen matrix until they are eliminated by recombination during annealing to still higher temperatures.^24^ The proton can originate in pre-organized proton-delivery network(s) within the active site, as observed in cryoreduction studies of oxygenated heme enzymes where we have shown that proton transfer can occur even at liquid helium temperatures (4–7 K),^36^ while without such pre-organization the proton transfer requires activation at temperatures well above 77 K.^34,39^

**Scheme 2:**
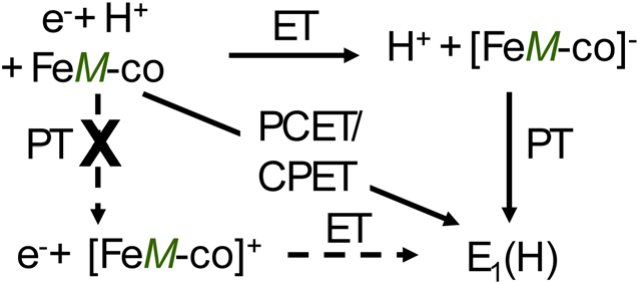
A ‘square-scheme’ representation of alternative mechanisms for generation of E_1_(H) by cryoreduction of the E_0_ resting state cofactor, FeM-co; the electron is denoted e^−^(s), proton, H^+^, to emphasize their different origins.

A process involving both PT and ET is typically discussed within a ‘square-scheme’^23,26,30,40^ that captures the three ways to transfer both an electron and a proton. This is illustrated in Scheme 2, but with direct application to nitrogenase as further discussed in the following paragraphs: proton-transfer (PT) first, then electron transfer (ET), with the process denoted, PT/ET; electron transfer first, followed by proton transfer in the frozen solution, ET/PT; or when the two processes are coupled and occur concurrently, proton-coupled electron transfer (PCET). However, this ‘diagonal’ pathway, in which an [e^−^/H^+^] are transferred without formation of a relatively stable intermediate at one of the ‘antidiagonal corners’, can occur by many pathways (*e.g.*, early/late transition state). Indeed, this process recently has been further organized into a ‘PCET reactivity continuum’.^28^ At one extreme is hydrogen atom transfer (HAT), the concerted transfer of a hydrogen atom; at the other, represented by this study of nitrogenase, the e^−^ and H^+^ transfer from spatially distinct sites, denoted multiple-site coupled (or even concerted) proton-electron transfer (MS-CPET). Thus, to quote an early review by Mayer and Rhile,^30^ “There are almost as many definitions of ‘‘proton-coupled electron transfer’’ (PCET) as there are groups working in this area”. To begin, therefore, it is important to clarify how delivery of an [e^−^/H^+^] to nitrogenase through cryoreduction/annealing maps onto current formulations of electron/proton transfer, and what is meant here when we use the shorthand term, PCET.

As illustrated in Scheme 2, the present experiments are conveniently discussed in terms of four states of the cofactor. The initial cofactor state under consideration is that of the as-isolated E_0_ resting state, denoted simply as, FeM-co. During the 77 K irradiation period, mobile irradiation-generated electrons in the solvent are capable of cofactor reduction (ET), while during subsequent cryoannealing at higher temperatures, persistent radicals generated in the solvent can act as reductant; both are denoted in Scheme 2 as e^−^. The proton ultimately transferred to the catalytic cofactor, denoted, H^+^, may be delivered by a preorganized proton-delivery network,^39^ or may be activated during cryoannealing at higher temperatures, typically at or above ∼200 K.^41^ Thus, as emphasized in Scheme 2, this reaction can be described as a situation where the e^−^ and H^+^ transfer from distinct ‘reagents’, “multi-site coupled proton-electron transfer”, MS-CPET,^28^ although for convenience we retain the most-commonly used generic term, PCET.

Within the physiological pH range, the FeM-cofactors cannot ‘simply’ accept a proton, and this is unchanged by the irradiation process, which does not increase the proton activity, so initial proton transfer to the cofactor (PT) is precluded, therefore precluding the PT/ET pathway, in effect collapsing the ‘square-scheme’ into a ‘triangle-scheme’ with only two pathways for [e^−^/H^+^] delivery (Scheme 2): either the electron reduces the cofactor to produce a reduced intermediate, and this is followed by protonation of the cofactor to give the product state (ET/PT), or the electron can only transfer if it is accompanied by the proton, namely when coupled to the proton transfer. However, as one can only monitor the states at the corners of the square during cryoreduction/annealing, not the kinetics of transformation among them, as noted above, we do not address finer details of a PCET/CPET process.

### Cryoreduction/annealing of MoFe protein

The present observations build on the results of earlier cryoreduction/annealing studies of the E_0_ → E_1_(H) process in MoFe protein. An early Mössbauer study had shown that the equilibrium E_1_(H) state formed and freeze-trapped during turnover with the electron-delivery component of nitrogenases (Fe protein) contains an integer-spin reduced FeMo-co (denoted M^R^ (*S* ≥ 1)) without a major change in spin coupling among the Fe ions.^18^ However, 77 K cryoreduction of the MoFe-protein E_0_ causes electron transfer (ET) to the FeMo-cofactor that produces an alternative, nonequilibrium reduced FeMo-co state (M^I^), with a major change in spin coupling.^18^ Subsequently, an X-ray absorption/Mossbauer study^19^ disclosed a conversion of M^I^ to the M^R^ of E_1_(H) upon annealing at 200 K, indicating that proton transfer to the reduced M^I^ cofactor is only enabled by thermodynamic or conformational activation, thus overall generating FeMo-co in the E_1_(H) state through an ET/PT process: reduction of E_0_ to M^I^ followed by protonation of M^I^ to form M^R^.

Subsequently, we showed^24^ that 77 K cryoreduction of equilibrium E_1_(H), accumulated during low-flux turnover with Fe protein and freeze-trapped, directly produced the E_2_(2H) state. As the transformation occurred during the 77 K radiolysis, it was not, however, possible to establish whether the E_1_(H) → E_2_(2H) conversion also involves sequential electron and proton delivery (ET/PT), or coupled delivery of a proton and an electron (PCET), although the findings for E_0_ → E_1_(H) suggest the former.

**Fig. 2:**
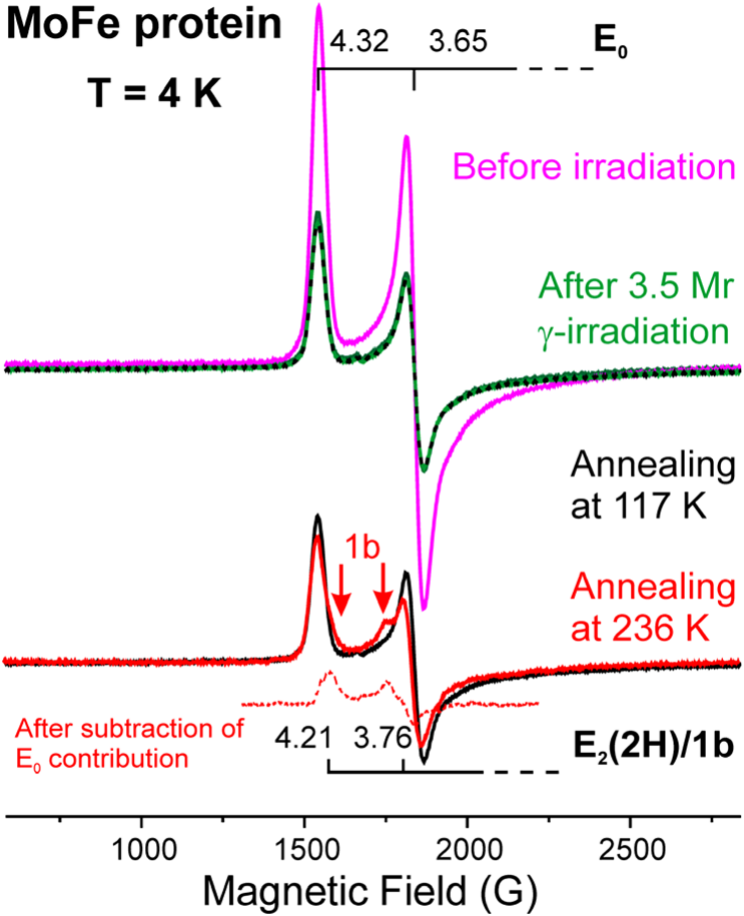
4 K X-band EPR spectra of MoFe-protein (MoFe) before and after cryoreduction (3.5 Mrad) and after subsequent annealing at 117 K and 236 K, 2 minutes each, with a subtraction revealing the 1b/E_2_(2H) contribution.

Fig. 2 shows the low-field portion of the 4 K, X-band EPR spectrum of MoFe-protein, with features from the *S* = 3/2 FeMo-co E_0_ resting state at *g*_1_ = 4.32, *g*_2_ = 3.65. As shown, cryoreduction at 77 K with 3.5 Mrad of γ-radiation reduces ∼50% of the E_0_ state. As reduction of the EPR-active E_0_ state by one electron must produce a diamagnetic or integer-spin product, as expected the reduction does not generate a new EPR signal from the cofactor, while generating strong *g* ∼2 signals from radicals formed by the gamma irradiation of the solvent. Annealing of γ-irradiated MoFe-protein for two minutes at 117 K causes no further change in the intensity of the E_0_ signal, Fig. 2, while leaving the reduced cofactor in the M^I^ form, as shown by the previous studies.^18,19^ However, somewhat surprisingly, subsequent annealing for two minutes at 236 K causes both a further, albeit small, reduction of E_0_ and the appearance of a weak signal from the E_2_(2H) state, denoted 1b, with *g*_1_ = 4.21, *g*_2_ = 3.76 (ref. 42) as revealed by subtraction of the E_0_ contribution in Fig. 2. The absence of any new signal upon 77 K radiolysis shows that the 1b signal does not arise from a double-reduction of E_0_ by the mobile electrons, and thus the appearance of 1b during 236 K annealing shows that at 236 K the radiation-generated radicals are capable of reducing the E_1_(H) that had been formed by ET/PT, with follow-up, activated acquisition of the second proton at 236 K producing E_2_(2H) (ET/PT). Thus, the essential finding from the prior studies as complemented by the present observations is that the E_0_ → E_1_(H) process involves ET/PT, while the observations here with MoFe further suggest that at 236 K the radiation-generated solvent radicals also can carry out E_1_(H) → E_2_(2H) by ET/PT. These findings set the stage for considerations of the cryoreduction/annealing of the other two nitrogenases, the VFe- and FeFe-proteins.

### Cryoreduction/annealing of VFe-protein

**Fig. 3:**
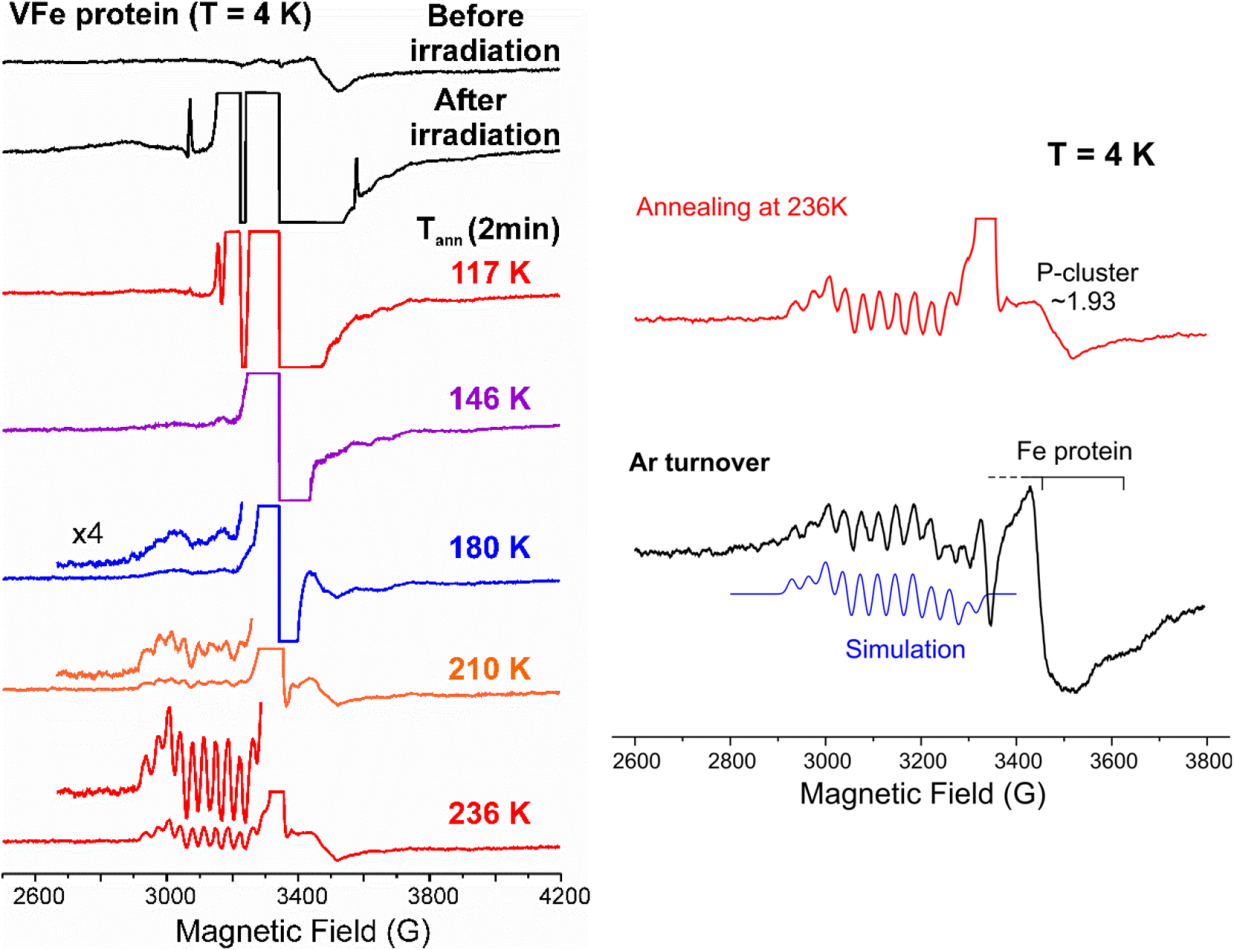
4 K X-band EPR spectra of VFe-protein, *g*-2 region. Left: before and after γ-irradiation (3.5 Mrad) and subsequent sequential annealing at 117 K, 146 K, 180 K, 210 K, and 236 K for 2 minutes each. For 180 K, 210 K and 236 K annealing, a 4x-expanded spectrum is included. Right: (upper) background-subtracted, scan after cryoreduction and annealing at 236 K; (lower) spectrum of VFe-protein freeze-quenched during catalytic turnover with Fe protein under Ar, and containing a signal from the catalytic intermediate previously designated E_1,3_(1,3H). Simulation: EasySpin using *g* = [2.18, 2.12, 2.09] and ^51^V (*I* = 7/2) hyperfine coupling, *a*_iso_ = 110 MHz.

It has been shown that FeV-co in the as-isolated, resting state (E_0_) VFe-protein is EPR-silent, being either *S* = 0 or *S* = 1,^15^ and additional results supporting that assignment are presented in SI. Thus, the low-field features in a full-field 4 K, X-band EPR spectrum of as-isolated VFe-protein (Fig. S1) do not arise from catalytically active FeV-co and henceforth will be mostly ignored. The *g* ∼1.93 signal seen in the expanded *g*-2 region of the spectra, Fig. 3, left, collected with a fine grid of cryoannealing stages is associated with forms of the P-cluster.^15^

If the mobile electrons ejected from solvent during 3.5 Mrad 77 K γ-irradiation of EPR-silent E_0_ VFe-protein were able to generate the one-electron reduced FeV-co, as it does with FeMo-co, this must produce an EPR-visible, *S* = 1/2 or 3/2 FeV-co E_1_ signal, with *S* = 3/2 being strongly supported by experiments in which reduced FeV-co was loaded onto the protein NifX,^43^ but neither type of signal appears, Fig. 3 and S1. The only change in the EPR spectrum, Fig. 3, left, is the appearance of strong *g* ∼2 signals from radiation-generated solvent radicals, which hide any change at *g* ∼1.93, and of a low-intensity, sharp doublet from H-atoms, likely produced in the quartz tube by the irradiation (plus a decrease of the catalytically irrelevant high-*g* signals by ∼50%, Fig. S1). In short, unlike MoFe-protein, the mobile electrons formed during 77 K γ-irradiation of VFe-protein are unable to reduce the EPR-silent FeV-co E_0_ resting state, which would have necessarily produced an EPR-active product of reduction.

Annealing of γ-irradiated VFe-protein for two minutes at 117 K causes typical cryoannealing decreases to the radical signals, which partially uncovers the *g*-1.93 signal, and a loss of the H-atom doublet, but introduces no new signal, in particular none with ^51^V hyperfine splittings, indication that there has been no reduction of FeV-co at this temperature, Fig. 3, left. Likewise, upon 2 min annealing at 146 K the radical signals decrease further without the appearance of new signals. Annealing at 180 K further decreases the radical signals, while slightly uncovering the *g*-1.93 signal. However, the 4-fold blowup of that spectrum in Fig. 3, left appears to give the faintest hint of the formation of signal with resolved ^51^V hyperfine splittings. The blow-up of the spectrum obtained after further annealing at 210 K for 2 min (Fig. 3, left) clearly shows the appearance of an *S* = 1/2 signal that, though weak, unambiguously displays the resolved ^51^V hyperfine splitting characteristic of the catalytic intermediate previously trapped during turnover of VFe-protein.^15^ This signal is greatly enhanced and perhaps somewhat sharpened by further annealing at 236 K for 2 min (Fig. 3, left), with no further increase upon two additional minutes of annealing at this temperature. This signal is simulated (Fig. 3, right) with parameters that establish its identification as the intermediate freeze-trapped during catalytic turnover, *g* = [2.18, 2.12, 2.09] and well-defined, isotropic ^51^V (*I* = 7/2) hyperfine splittings, *a*_iso_ = 110 MHz.^15^ Given its EPR visibility, this catalytic intermediate must be an early E_*n*_(*n*H) state, *n* = 1, 3. As noted above, in the prior work this species was designated E_1,3_(1,3H) because the turnover experiments did not definitively determine whether *n* = 1 or 3. However, as the reduction during annealing is caused by the limited number of radicals remaining at *T* ≳ 210 K, and this signal is the first new one to appear during annealing, it clearly must arise by delivery of a single electron and proton to VFe-protein E_0_, not three, to form what is thus recognized as the E_1_(H) state.

As the EPR-silent E_0_ FeV-co is not reduced to an EPR-active state by the strongly reducing, mobile electrons generated during the 77 K γ-irradiation, but only upon annealing, we conclude that FeV-cofactor reduction is only enabled by delivery of a proton together with the electron, PCET, but that, like MoFe protein, the VFe-protein is incapable of delivering that proton at 77 K. Instead, the appearance of the E_1_(H) state in VFe-protein only after annealing at and above ∼210 K (Fig. 3, left), establishes that the proton transfer to FeV-co requires activation through annealing to such temperatures.

**Scheme 3:**
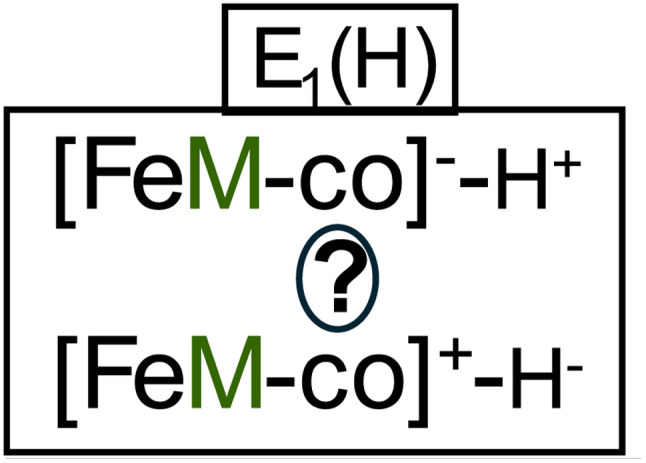
Possible states of E_1_(H): top, reduced cofactor, sulfur-bound proton; bottom, cofactor with Fe-hydride, thus formally oxidized.

What is the nature of the FeV-co E_1_(H) cofactor: is it a reduced cofactor with a proton presumably bound to a sulfide, or a formally oxidized cofactor with an Fe-bound hydride (Scheme 3)? This was tested by carrying out intracavity 12 K 450 nm photolysis, which previously had shown that E_1_(H) of FeFe-protein indeed is hydride-bound,^17^ and as a result, that the FeFe-co metal-ion core is formally described as being one-electron oxidized relative to the resting state. It was proposed that this behavior applies to all three nitrogenase isozymes,^17^ and we have tested this for VFe-protein. When VFe-protein sample exhibiting the E_1_(H) signal is cooled to 12 K and photolyzed with 450 nm light for 20 minutes, the E_1_(H) signal decreases slightly (Fig. S3), a change attributable to hydride isomerization, just as seen for the hydride bound in to FeFe-co in E_1_(H) of the FeFe-protein.^17^ For FeV-co, the photo-generated isomer is not detected, likely because its signal is too weak. A further 20 min of photolysis caused no further change (Fig. S3). We interpret this as indicating that the photoisomer also is photolabile, as is the case hydride-bound E_1_(H) isormers of FeFe-co, and the resulting photostationary state slightly favors E_1_(H). Regardless, the photolysis change in the signal of the FeV-co E_1_(H) state indicates that it too is hydride-bound.

### Cryoreduction/annealing of FeFe-protein

The FeFe-protein was the first of the three FeM-proteins for which the magnetic properties of its catalytic FeFe-co had been characterized in the E_1_(H) state: E_0_ is EPR-silent (diamagnetic or integer spin) and E_1_(H) has S = 1/2, with *g*-values, *g* = [1.965, 1.928, 1.779].^16^ The E_1_(H) FeFe-co was shown to contain a metal-bound hydride by the finding that 450 nm photolysis converts the E_1_(H) to a state denoted E_1_(H)*, *g* = [2.009, 1.950, 1.860], with a different mode of hydride binding.^17^

**Fig. 4:**
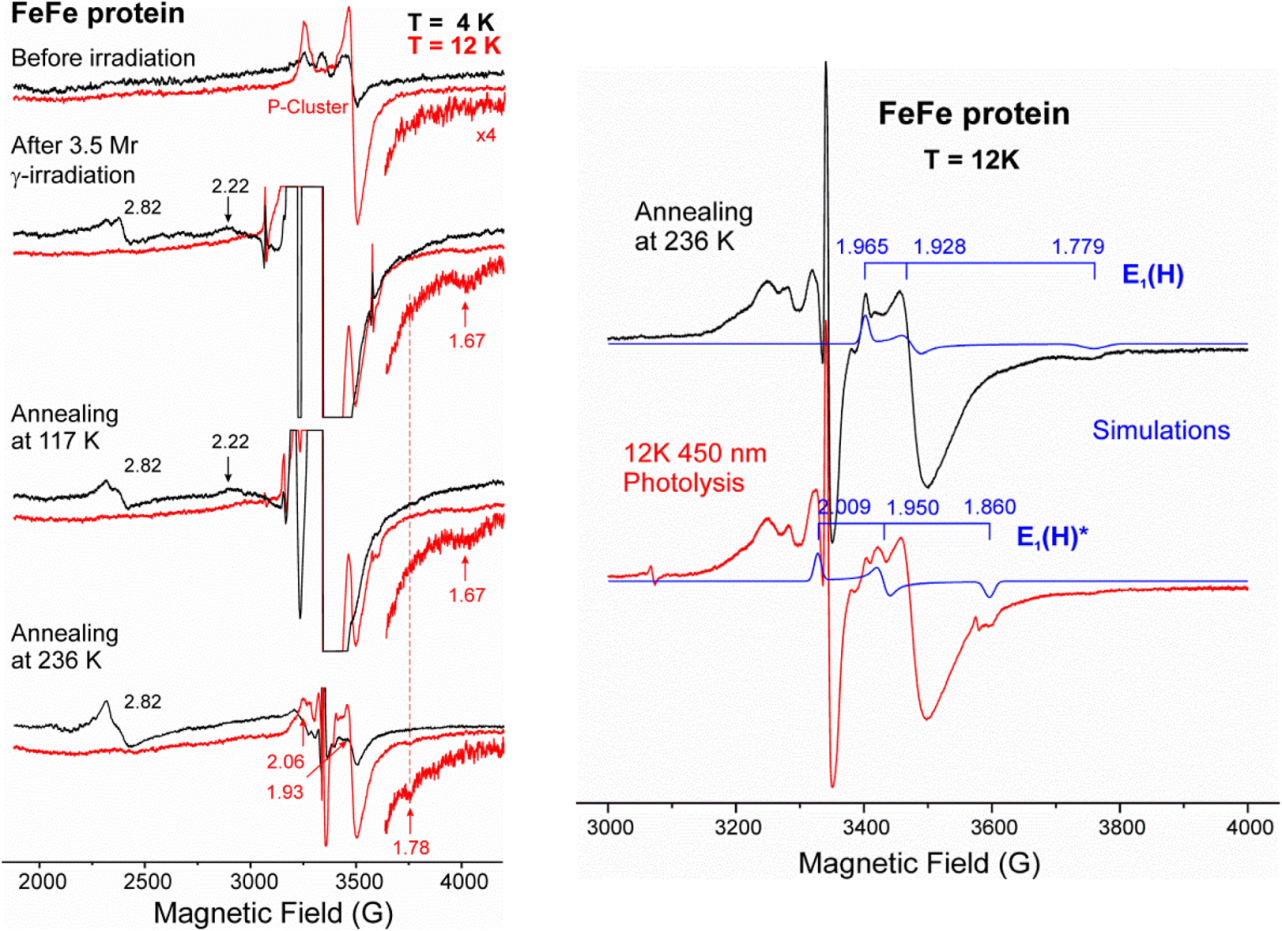
X-band EPR spectra of FeFe-protein. Left: spectra at 4 K and 12 K taken before and after cryoreduction (3.5 Mrad), and then after subsequent annealing at 117 K and 236 K for 2 minutes each. Of particular note are the weak *g*_‖_-type feature at *g* = 1.67 generated by reduction, which persists after 117 K annealing but converts to a *g* = 1.78 feature upon 236 K annealing. Right: (upper) expansion of 12 K spectrum after 236 K annealing, with superimposed simulation for E_1_(H) with the displayed *g*-values. (Lower) 12 K spectrum after subsequent 450 nm photolysis (20 min) with superimposed simulation for E_1_(H)* with indicated g-values (the extremely weak H-atom signal produced by photolysis is likely in the quartz tube).

Fig. 4, left shows the 4 K and 12 K X-band spectra of the EPR-silent E_0_ resting-state of FeFe-protein. Neither the low-field features (Fig. S1), nor the *g* ∼2 features in Fig. 4, left, arise from catalytically active FeFe-co, and the *g* ∼1.93 signal is associated with the oxidized P-cluster. The 4 K spectrum taken after 77 K γ-irradiation of the FeFe-protein shows that the radiation produces a relatively-strong metal-cluster signal at *g* ∼2.82 that is attributable to the reduction of previously unrecognized EPR-silent cluster species that accompany those giving rise to the spurious low-field signals (Fig. S1), an additional *S* = 1/2 cluster-type signal at *g* ∼2.22, as well as an H-atom doublet. In the post-irradiation spectrum taken at 12 K, faster spin-relaxation causes both the irradiation-generated cluster signals to disappear. Upon subsequent 117 K annealing, all of the cluster-like signals seen at 4 K after 77 K γ-irradiation persist, with slight annealing-induced relaxation of the *g* ∼2.22 center. Upon 236 K annealing, the *g* ∼2.82 signal observable at 4 K is seen to undergo further structural/spectroscopic relaxation, and features associated with oxidized P-cluster are revealed, at *g* = 2.06, 1.93; these increase in intensity at 12 K, as expected because this spectrum was partially saturated at 4 K.

Importantly, the 77 K cryoreduction also has created an *S* = 1/2 signal that is slowly relaxing, like E_1_(H), and with *g*_3_ = 1.67 similar to that of E_1_(H) (*g*_3_ = 1.78).^17^ The 12 K spectrum then taken after 117 K annealing retains the *g*_3_ = 1.67 signal, perhaps somewhat broadened by conformational relaxation. However, upon 236 K annealing, the *g*_3_ = 1.67 feature converts to the *g*_3_ = 1.78 feature of the E_1_(H) spectrum, whose *g*_1_ = 1.96 feature also is seen, Fig. 4, left. As previously reported, and reproduced in Fig. 4, right, and Fig. S3, upon 450 nm photolysis of the hydride-bound E_1_(H) it converts into a hydride isomer, E_1_(H)*, also with a distinct but similar value, *g*_3_ = 1.86 (with loss of the E_1_(H) *g*_1_ = 1.96 feature) The obvious interpretation of these observations is that each of the three of these ‘low-*g*_3_’ signals arises from an isomer of E_1_(H), and that at 77 K the electrons produced by γ-irradiation of E_0_ FeFe-co produce an isomer of E_1_(H) with *g*_3_ = 1.67, which we denote E_1_(H)^#^, through the transfer of an electron accompanied by a proton from a pre-organized donor within the active-site cavity. This isomer then relaxes to E_1_(H) upon annealing at 236 K, a behavior analogous to the relaxation of E_1_(H)* to E_1_(H) observed at temperatures above 145 K.^17^ These observations show that during 77 K γ-irradiation E_0_ FeFe-co acquires both a mobile electron and a proton to produce the E_1_(H)^#^ isomer of E_1_(H) with *g*_3_ = 1.67.

The conclusion that 77 K cryoreduction of the E_0_ state of FeFe-protein directly produces an E_1_(H) isomer implies that FeFe-protein contains a functional, pre-organized proton-delivery network able to transfer the proton along with the irradiation-generated electron. The ability of FeFe-protein to carry out PT at 77 K contrasts with the behavior both of MoFe protein, where proton delivery (PT) only was observed at ∼200 K, and of VFe-protein, where PCET is observed, but only upon activated proton transfer at *T* ≳ 200 K. This difference indicates that the MoFe and VFe-proteins lack a pre-organized network for proton-delivery at 77 K, in contrast to FeFe protein, and that proton delivery in those two requires activation during annealing at higher temperatures. Formation of the *g*_3_ = 1.67 E_1_(H)^#^ state of FeFe protein during 77 K cryoreduction suggests that this occurs by PCET, but does not preclude the possibility that the proton transfer occurs subsequent to electron transfer, namely ET/PT at 77 K.

## Summary and conclusions

This report has expanded our understanding of nitrogen fixation's biochemical intricacies by providing insights into multiple issues associated with electron and proton delivery to the active sites of the three MFe-proteins during the E_0_ → E_1_(H) transformation.

First, for MoFe- and VFe-proteins, proton delivery can only occur through activation during cryoannealing at ∼200 K and above, but for FeFe-protein proton delivery occurs during 77 K cryoreduction, indicating the presence of a preorganized protein-delivery network in FeFe-protein. This difference parallels the dichotomy found in the O_2_-activating enzymes, with pre-formed, proton-delivery networks present in cytochrome P450cam and heme oxygenase,^35,36^ but only activated proton-delivery in nitric-oxide synthase.^34^ Examination of the nitrogenase crystal structures^1,2^ has identified potential proton-donating residues within the active site.^44–47^ However, given the complexity of the nitrogenase structures, and indeed the differences among the cofactors themselves (Fig. 1), more detailed attempts to analyze the structural basis for proton delivery to the catalytic cofactors is beyond the scope of this report.

**Fig. 5:**
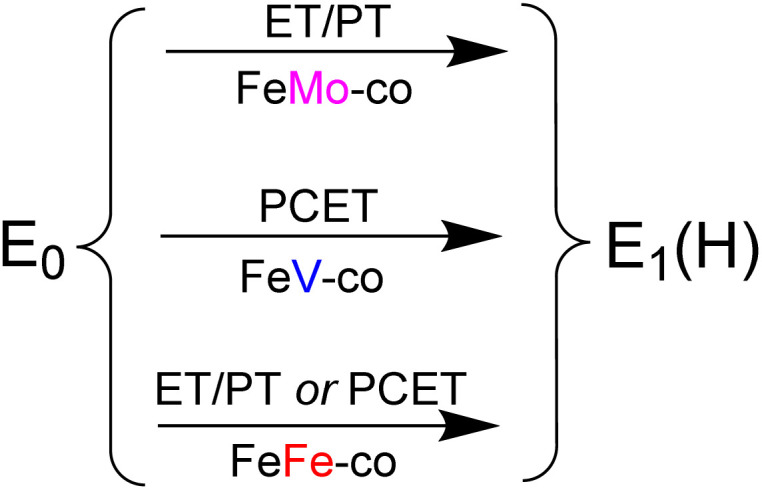
Summary of electron/proton delivery to the three FeM-cofactors (FeM-co).

Second, as a further distinction among the nitrogenases, 77 K cryoreduction/annealing experiments show that [e^−^/H^+^] delivery occurs by ET/PT for FeMo-co, but through proton-coupled electron transfer, PCET, for FeV-co, while the two alternatives cannot be distinguished for FeFe-co, as summarized in Fig. 5. Future Mossbauer^18^ and/or X-ray spectroscopic measurements^19^ would distinguish these two possibilities for FeFe-co, as they have done for FeMo-co.

In addition, we have clarified the identity of the intermediate previously trapped during turnover of VFe-protein, and denoted E_1,3_(1,3H) because it could not with certainty be assigned to the *n* = 1 or 3 state:^15^ we now find that *n* = 1; this signal (Fig. 3) belongs to E_1_(H).

Finally, 450 nm photolysis of the EPR-visible E_1_(H) intermediate of FeV-co, as well as that of FeFe-co,^17^ establishes that both contain a hydride bound to a formally oxidized FeM-co, and adds support to our previous suggestion that this is likely true for all three isozymes.

During catalytic nitrogenase turnover with the Fe protein at ambient temperatures, the actual delivery of an electron to FeM-cofactor is an extraordinarily complex process, which for MoFe-protein has been shown to (i) first involve conformational gating attributable to large-scale motions within the Fe protein-MoFe protein complex;^20^ (ii) exhibit intra-MoFe-protein ‘deficit spending’ in which the P-cluster donates an electron to FeMo-co before the P-cluster accepts the electron being delivered by the Fe protein;^21^ (iii) and with evidence that the two halves of the MoFe protein exhibit ‘negative cooperativity’/‘half-sites reactivity’.^22^

Cryoreduction of MFe-proteins alone in the frozen state does not capture many of those complexities of the catalytic E_0_ → E_1_(H) step. However, EPR-visible intermediates formed during Fe protein-free cryoreduction/annealing – E_1_ for FeFe and VFe, E_2_ for MoFe– are identical to those trapped during catalytic turnover with electron delivery by Fe protein. This supports the interpretation that in the present experiments a FeM-cofactor accepts an [e^−^/H^+^] by a process that reflects core features of the reactions that occur during turnover with the Fe protein at room temperature.

As concluding remarks, we have shown that all three isozymes exhibit the critical reductive-elimination/oxidative addition (re/oa) catalytic mechanism illustrated in Scheme 1, in which N_2_ binding/reduction is driven by the concerted reductive elimination of H_2_.^4^ However, the catalytic activities of the three isozymes nonetheless differ, with MoFe-protein most active for N_2_ reduction and FeFe-protein least,^4^ and the catalytic reduction of other substrates differs sharply among the three.^1^ The mechanistic differences observed here provide a first step towards revealing the source of such catalytic differences.

## Author contributions

R. D. oversaw cryoreduction/annealing procedures; D. A. L, performed EPR measurements; D. F. H. prepared samples; D. R. D. and L. C. S oversaw sample preparation; B. M. H., along with L. C. S., formulated the approach, all authors contributed to writing the manuscript.

## Conflicts of interest

The authors declare no competing financial interest.

## Supplementary Material

SC-016-D5SC05488E-s001

## Data Availability

Spectra and simulations are available from the authors upon request. Supplementary information is available. See DOI: https://doi.org/10.1039/d5sc05488e.
