# Supplementary material for: Proton transfer during reduction of the catalytic metallo-cofactors of the three nitrogenase isozymes
Source: Chem Sci. 2025 Sep 12;16(40):18729–38. doi: 10.1039/d5sc05488e (PMC12429461; doi:10.1039/d5sc05488e)
Supplement: SC-016-D5SC05488E-s001 [file SC-016-D5SC05488E-s001.pdf]

Supplementary Information (SI) for Chemical Science.
This journal is © The Royal Society of Chemistry 2025

Supplementary Information

Proton Transfer During Reduction of the Catalytic Metallo-Cofactors of the Three Nitrogenase Isozymes

Roman Davydov[1], Dmitriy A. Lukoyanov[1], Derek F. Harris[2], Dennis R. Dean[3], Lance C. Seefeldt[2*], Brian M. Hoffman[1*]

[1]Department of Chemistry, Northwestern University, 2145 Sheridan Road, Evanston, Illinois 60208
[2] Department of Chemistry and Biochemistry, Utah State University, Logan, Utah, 84322.
[3] Department of Biochemistry, Virginia Polytechnic Institute and State University, Blacksburg, Virginia
24061

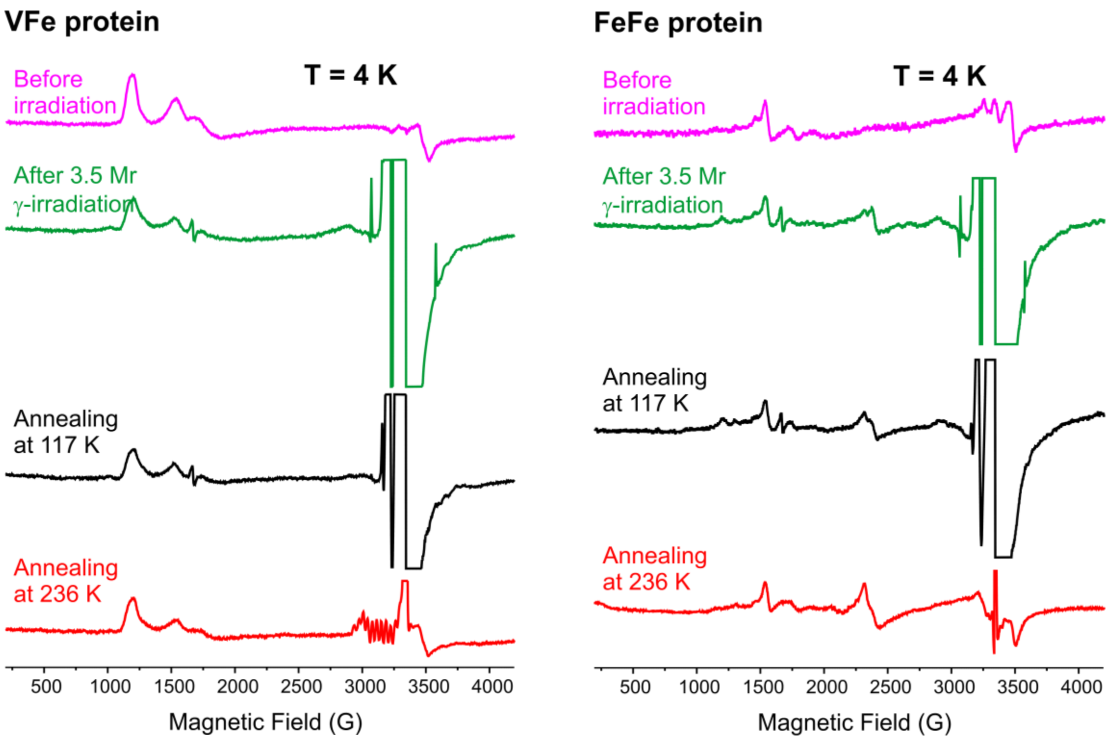

Fig S1. 4 K Full-field, X-band EPR spectra of VFe-protein (left) and FeFe-protein (right). Before and after γ-irradiation (3.5 Mrad) and subsequent annealing at 117 K and 236 K for 2 minutes each. EPR spectra were recorded on X-band Bruker ESP-300 spectrometer equipped with Oxford Instruments ESR 900 continuous liquid He flow cryostat; conditions in all spectra, microwave frequency, ν_{MW} = 9.364 GHz; Mod Amp = 10 G.

Confirmation that resting state of VFe-protein is EPR-Silent.

We tested the assignment of the resting state (E_0) of the VFe-protein as being EPR-silent, and the S = ½ hyperfine (HF)-split catalytic intermediate as the E_1 state, rather than the resting state being EPR-active with its signal associated with the low-field g ~ 5.6-4.0 signals of as-isolated enzyme. In this second case, the S = ½ HF-split state would be an E_2 state that unusually has acquired two electrons, rather than one, during cryoreduction and annealing at 236 K. As the test, we took the enzyme that had been cryoreduced and annealed for 2 min at 236 K and further annealed it, first at 223 K and then at 236 K (**Fig S2**).

The annealing at 223 K for 10 min caused no change in the EPR spectrum except for a typical decrease in the radical signal at g-2, **Fig S2, left**. The stability of the HF-split signal is consistent with it being associated with an E_1 state, which cannot relax to E_0 with loss of H_2. Additional annealing at 236 K for 5 min does cause a modest decrease in the S = ½ signal from the catalytic intermediate, but without the appearance of *any* new signal, or other change anywhere in the spectrum except for a further loss of radical signal; another 20 min of annealing at 236 K causes major loss of the intermediate, again with no other change but to the radical signal. In particular, as shown in **Fig S2, right**, the major loss in the S = ½ signal during the annealing process is *not* accompanied by *any* increase in the intensity of the low-field signals (between ~1,200 and 1750 G; g ~ 5.6-4.0).

If one imagined the S = ½ signal were associated with an EPR-active E_2 state, with the resting state therefore EPR-active and spectroscopically visible among the low-field g ~ 5.6-4.0 signals, then the expectation from studies of the annealing of MoFe intermediates would be that decay of the S = ½ signal would occur through loss of H_2 and conversion to the EPR-active E_0, with a necessary accompanying increase of its signal in the low-field region. *But, absolutely no such change occurs,* as can be seen in **Fig S2, right**, *and as stated, no new signal of any kind appears* in parallel with the loss of the S = ½ response **Fig S2, left**.

These observations support the view that E_0 is EPR-silent, not associated with the g ~ 5.6-4.0 signals, and that the S =1/2 signal is not an E_2 state. In the main text, we in fact assign the S = ½ signal to the E_1 state of VFe protein, and the resting E_0 state as EPR-silent. An E_1 state cannot relax to E_0 through loss of H_2, then why does the S = ½ signal decay during the 236 K annealing (**Fig S2**)? We believe that at 236 K the remaining radicals generated by the cryoreduction are able to reduce the E_1 state to an EPR-silent E_2 state, just as they were able to generate the HF-split signal from E_0; then, as seen in studies of the annealing of MoFe intermediates, this E_2 would relax to the EPR-silent E_0 through direct loss of H_2, which explains the absence of any other changes in the EPR spectrum that accompany the loss of the signal of the S = ½ intermediate.

In short, the observations of **Fig S2** strongly indicate that the VFe protein S = ½, hyperfine-split signal is indeed the singly-reduced E_1(H) state, and that the E_0 resting state is EPR-silent.

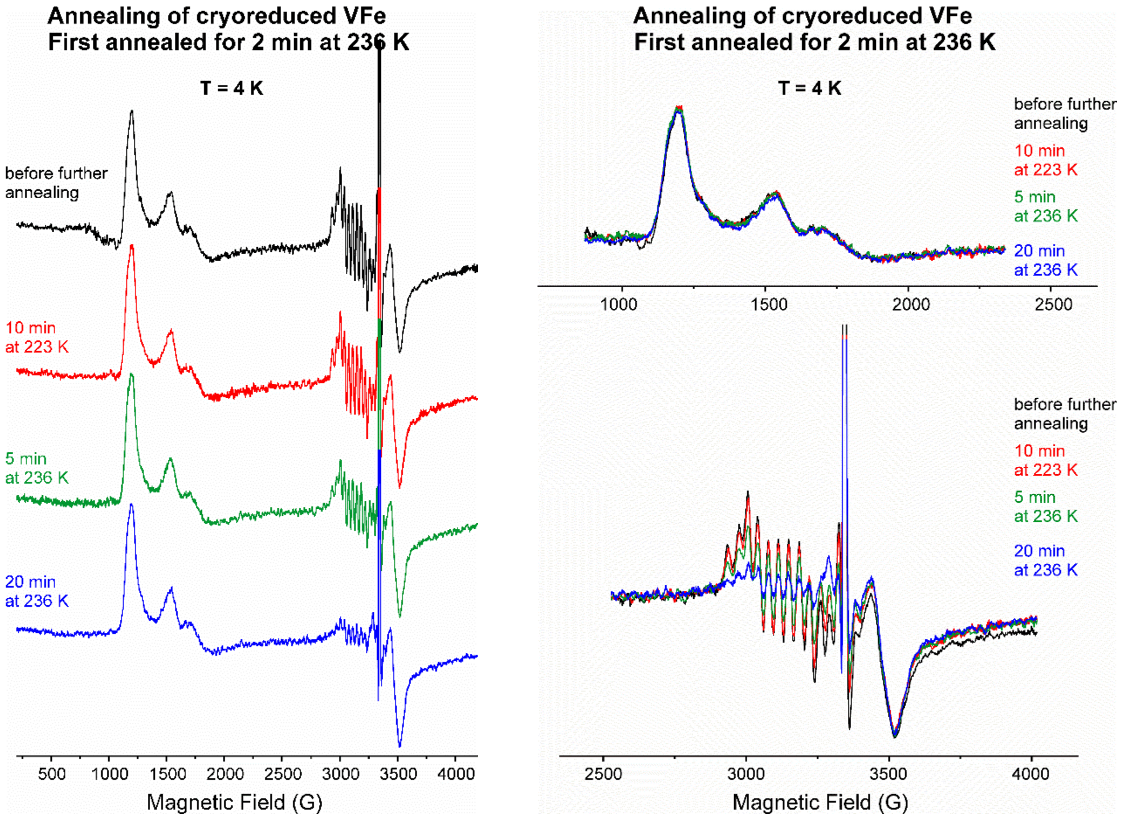

Fig S2: (*left*) X-band spectra of VFe protein that had been cryoreduced and annealed at 236 K for 2min, then subjected to further annealing as indicated. (*right*) Overlap of low-field (upper) and high-field (lower) portions of the spectra. *Conditions:* MW frequency, 9.364 GHz, Mod Amp, 8 G, T = 4K.

Photolysis of $E_1(H)$ intermediates formed in cryoreduced VFe and FeFe proteins by annealing at 236 K.

The $E_1(H)$ intermediate of cryoreduced and 236 K annealed VFe protein was tested with 12 K 450 nm photolysis, which reveals the presence of a bound hydride by causing its isomerization, as was previously shown for $E_1(H)$ of FeFe and $E_2(2H)$ of MoFe, states with a single bound hydride.(refs 8, 13 main text). Upon 20 minutes intracavity photolysis the $E_1(H)$ signal of VFe decreased by ~ 20%, as demonstrated in the difference spectrum (**Fig S3, left**), without a detectable signal from the photo-generated state. Additional 20 minutes of photolysis did not induce farther decrease of the VFe $E_1(H)$ signal. In contrast, the similar experiment with $E_2(2H)$ of MoFe (ref 8), mentioned in the main text, demonstrated ~70% loss of this photo-reactive state. As noted in the main text, we conclude that the photo-produced isomer also is photo-reactive, and that during first 20 minutes of photolysis a photo-stationary state was established between initial $E_1(H)$ and its photoinduced isomer, and that the latter has a somewhat higher photolability, and so does not accumulate enough during photolysis to be detected.

While photolysis of VFe $E_1(H)$ was performed to characterize the state itself, photolysis of FeFe sample was applied to confirm that the weak signal formed by cryoreduction and 236 K annealing is really from an isomer of the FeFe $E_1(H)$ intermediate that forms during turnover, and which was previously well characterized by EPR and 450 nm irradiation at cryogenic temperatures (ref 13 main text). Subtraction of FeFe spectra obtained before and after photolysis eliminates all photo-persistent signals, which dominate the direct spectra, leaving only features of photolabile states **(Fig S3, right).** The resulting difference trace can be well described as difference of EPR signals from $E_1(H)$ state and its photoinduced isomer $E_1(H)^*$ thus confirming that weak signal formed by cryoreduction and 236 K annealing of FeFe protein is indeed from an $E_1(H)$ intermediate.

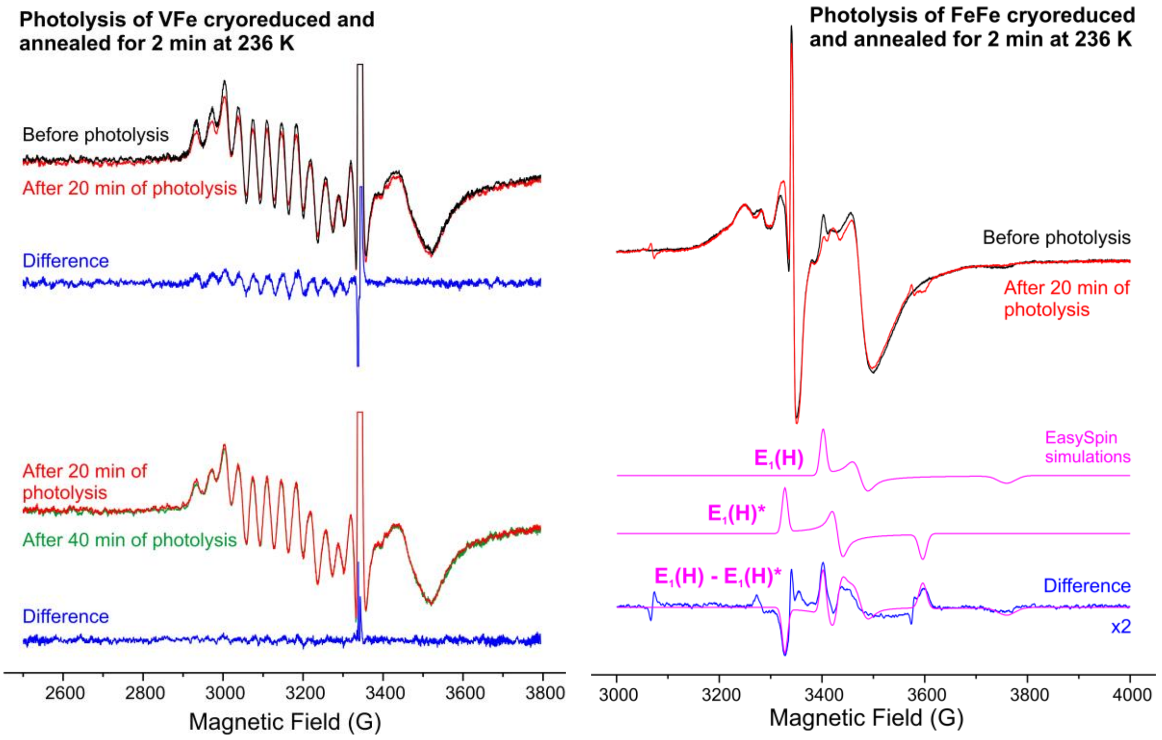

Fig S3: X-band EPR spectra of VFe (left) and FeFe (right) proteins that had been cryoreduced and annealed at 236 K for 2min, then subjected to 450 nm photolysis at 12 K. Weak H-atom signals detectable at 12 K and changes in the radical signal are likely photolysis-produced in the quartz tubes. *Conditions:* MW frequency, 9.364 GHz, Mod Amp, 5 G for VFe and 8 G for FeFe, T = 4 K for VFe and 12 K for FeFe.
